# Recruitment strategies to increase racial and ethnic diversity in anorexia nervosa clinical research

**DOI:** 10.1186/s40337-023-00844-6

**Published:** 2023-07-15

**Authors:** Julianne Strauch, Alexandra Agnew, Erinne Meenaghan, Karen K. Miller, Melanie S. Haines

**Affiliations:** 1 Neuroendocrine Unit, Massachusetts General Hospital, 50 Staniford Street, Suite 750B, Boston, MA 02114 USA; 2 Harvard Medical School, Boston, MA USA

**Keywords:** Anorexia nervosa, Bone health, Diversity, Equity, Inclusion, Community health, Social media

## Abstract

**Objective:**

Inclusion of underrepresented racial and ethnic groups in eating disorder (ED) research is a critical unmet need, but evidence-based recruitment strategies are lacking. We sought to determine whether methods we had implemented to increase recruitment of underrepresented racial and ethnic groups were successful in improving racial and ethnic diversity in a clinical trial in women with anorexia nervosa.

**Method:**

We implemented new strategies for recruitment of underrepresented racial and ethnic groups in a clinical trial on bone health in women with anorexia nervosa, including leveraging social media, liberalizing language on advertisements to be more inclusive of women who are as yet undiagnosed with the disorder or feel stigmatized by its name, translating advertisements to Spanish, and engaging community health centers. We compared participants’ race and ethnicity in this clinical trial versus two similar prior clinical trials.

**Results:**

The percent of non-White and Hispanic participants who have signed a consent form in our ongoing clinical trial (2021–2023) is higher versus two previous clinical trials on bone health in women with anorexia nervosa (2011–2019) with similar inclusion/exclusion criteria and endpoints [non-White: 11/38 (28.9%) vs. 11/188 (5.9%), Hispanic: 6/38 (15.8%) vs. 5/188 (2.7%), *p* ≤ 0.006]. There was no change in the percent of Black participants [0/38 (0%) vs. 1/188 (0.5%), *p* = 1.0].

**Discussion:**

Viewing clinical research recruitment through a diversity, equity, and inclusion lens can improve racial and ethnic diversity in ED research. Further research recruitment strategies are needed to be more inclusive of Black populations.

## Need for greater participant diversity in eating disorder research

Anorexia nervosa, an eating disorder associated with restrictive behaviors due to an intense fear of gaining weight and a distorted perception of body weight or shape, is often characterized as a disease of young, White, affluent women. However, anorexia nervosa can affect individuals of all genders, races, ages, socio-economic backgrounds, ethnicities, and sexual orientations [1–6]. Although eating disorder (ED) researchers acknowledge the importance of participant diversity in ED clinical studies to reflect the general population of children and adults who suffer from these diseases, racial and ethnic diversity continues to be a critical unmet need in ED research [1, 2, 7]. In a recent retrospective literature review, race and ethnicity data were reported in only 44% of published ED studies, of which approximately 72% of participants identified as White [7]. The authors of the retrospective literature review could not exclude reporting bias (i.e., that studies that were more racially and ethnically diverse were more likely to report race and ethnicity data compared to studies that were less diverse). Lack of inclusion of underrepresented racial and ethnic groups threatens the generalizability of research results, as different individuals may experience disease and respond to treatments differently, and sustains the misconception that EDs such as anorexia nervosa only affect young, White cis-gender females in affluent Western cultures [2, 7]. Additional personal and societal ramifications of not targeting underrepresented groups in research include contributing to racial and ethnic inequities in diagnosis, treatment and health outcomes. They may also contribute to mistrust of the medical and research community among groups that historically have been discriminated against and/or have been underserved by these institutions.

Several barriers exist to diagnosing and treating EDs in underrepresented racial and ethnic groups, as well as enrolling such groups in ED clinical research. Cultural differences surrounding mental health, diet, body ideals, and help-seeking behaviors may complicate disease presentation and result in underdiagnosis of EDs in populations that identify as non-White (i.e., American Indian or Alaska Native, Asian, Black or African American, Multiracial, Native Hawaiian or Other Pacific Islander) [1, 7]. Bias and underappreciation of the prevalence of EDs in non-White populations may further explain why individuals from underrepresented racial and ethnic groups are less likely to be screened for an ED and are less likely to be referred to a higher level of care following an ED diagnosis, even after controlling for symptom severity [3]. Underdiagnosis and undertreatment of EDs in non-White individuals contribute to the underrepresentation of such groups in ED research, as ED researchers often rely on referrals from clinical providers as a major recruitment source [8]. Limited accessibility to treatment services and research institutions as well as mistrust of the medical and research community pose additional barriers to both clinical care and participation in clinical studies in such populations [1, 2]. Despite the identification of such barriers, evidence-based strategies to increase recruitment of participants with diverse racial and ethnic backgrounds in ED research are lacking.

## Implementing new recruitment strategies to increase racial and ethnic diversity of anorexia nervosa research participants

Our clinical research team in the Neuroendocrine Unit at Massachusetts General Hospital is committed to improving treatments for low bone mineral density in anorexia nervosa, a common and severe co-morbidity associated with an increased risk of fractures [9]. Given that low bone mineral density is under-diagnosed among women with anorexia nervosa, a previous diagnosis of low bone mineral density is not required to consent to screen for our clinical research studies. Participants who signed a consent form in our two most recently completed bone health studies in women with anorexia nervosa were 90% White and 88% non-Hispanic (Table 1). In alignment with the need to improve diversity in ED research more broadly, we recognize that we must improve racial and ethnic diversity in our own research. We collaborated with the Community Access, Recruitment and Engagement Research Center at Massachusetts General Hospital to develop new approaches to recruit participants with more diverse racial and ethnic identities in our current clinical trial in women with anorexia nervosa and low bone density.

**Table 1:** Self-reported race and ethnicity data of participants who signed a consent form in two prior clinical trials versus a current clinical trial in women with anorexia nervosa and low bone mineral density

	Past clinical trials (n = 188)	Current clinical trial (n = 38)
*Race*		
American Indian or Alaska Native, (%)	1 (0.5%)	0
Asian, n (%)	7 (3.7%)	7 (18.4%)
Black or African American, n (%)	1 (0.5%)	0
Multiracial, n (%)	0	2 (5.3%)
Native Hawaiian or Other Pacific Islander, n (%)	0	0
Other, n (%)	2 (1.1%)	2 (5.3%)
White, n (%)	169 (89.9%)	27 (71.1%)
Declined, n (%)	0	0
Unknown, n (%)	8 (4.3%)	0
*Ethnicity*		
Hispanic or Latino, n (%)	5 (2.7%)	6 (15.8%)
Non-Hispanic or non-Latino, n (%)	165 (87.8%)	32 (84.2%)
Other, n (%)	0	0
Declined, n (%)	1 (0.5%)	0
Unknown, n (%)	17 (9.0%)	0

Self-identification outside of these categories was recorded as “other.” Race and ethnicity were recorded as “unknown” when self-reports were not obtained

In the past, we primarily recruited research participants through clinical referrals from a regional network of ED specialists and treatment programs, which may not reach individuals who have not yet been diagnosed with anorexia nervosa, have not yet sought treatment, or are unable to access ED specialists and treatment programs. We sought to overcome this limitation and engage more diverse communities in our research by advertising our study on publicly accessible platforms like social media, online recruitment sites, and university message boards. Additionally, we utilized the Mass General Brigham Research Patient Data Registry (RPDR), a centralized clinical data registry that gathers data from across the Mass General Brigham system, to identify and contact patients with anorexia nervosa across the system, including affiliate community health care centers. We also engaged and collaborated with trusted local community health centers that serve racially and ethnically diverse patient populations. At the suggestion of our community partners, we translated our study advertisements into Spanish and revised our recruitment materials to be more sensitive to cultural norms related to anorexia nervosa. For instance, our recruitment flyers now include images of women of diverse races and ethnicities. We liberalized the language in our study advertisements from “women with anorexia nervosa” to “women who are chronically underweight” or have been advised to take nutritional drinks as dietary supplements for weight gain. We made these changes to include women who may be undiagnosed with the disease or feel stigmatized by its name. When women respond to these flyers and consent to speak with our study team, our psychiatric nurse practitioner performs a clinical intake and assessment, resulting in new diagnoses of anorexia nervosa for some women who then enroll in our studies. We ask all participants for their self-reported race and ethnicity based on the categories listed in the Table 1. The percent of White participants randomized to active treatment or placebo in the two previous clinical trials has been previously reported with the results of the trials [10, 11]; race and ethnicity of individuals who consented to be screened for eligibility to participate in the trials has not previously been reported.

These new approaches to address inequities in our research have increased the racial and ethnic diversity of participants who signed a consent form in our current clinical trial compared to previous clinical trials on bone health in anorexia nervosa. The percent of non-White and Hispanic participants who signed a consent form (agreeing to be screened for eligibility) in our ongoing clinical trial to date (September 2021- February 2022) (n = 38) is significantly higher than that of our two previous clinical trials on bone health in women with anorexia nervosa (2011–2019) (n = 188) [10, 11] with similar inclusion/exclusion criteria and bone endpoints (non-White 28.9% vs 5.9%, *p* = 0.0002; Hispanic 15.8% vs 2.7%, *p* = 0.006) (Table 1). These differences remain significant among participants who completed a baseline study visit in the current clinical trial (n = 19) vs. two previous clinical trials (n = 112) (non-White 31.6% vs 7.1%, *p* = 0.007; Hispanic 21.1% vs 1.8%, *p* = 0.005). More than half (57.9%, n = 22) of participants who signed a consent form were recruited through public online platforms and social media, including at least half of non-White (63.6%, n = 7) and Hispanic (50.0%, n = 3) participants who signed a consent form. The other half of Hispanic participants were recruited from RPDR, while a smaller percent of the non-White participants (18.2%, n = 2) were recruited from RPDR. Race and ethnicity data were not obtained for 8 and 17 participants, respectively, who signed a consent form in one of the two previous studies. However, the percent of non-White and Hispanic participants who signed a consent form in the current clinical trial remains significantly higher than that of the previous clinical trial for which race and ethnicity data were obtained from all participants (n = 43) (non-White 28.9% vs 4.7%, *p* = 0.005; Hispanic 15.8% vs 2.3%, *p* = 0.047).

## Discussion

Our data demonstrate that viewing clinical research recruitment efforts through a diversity, equity, and inclusion lens can rapidly result in tangible improvements in the recruitment of racially and ethnically diverse participants in ED research. Our revised recruitment strategies have significantly increased the percent of non-White and Hispanic participants in our ongoing clinical trial compared to two previous clinical trials on bone health in women with anorexia nervosa. Expanding our recruitment efforts beyond clinical referrals to the general public through online and social media posts and the diverse patient populations treated at Mass General Brigham through the Research Patient Data Registry, screening participants who may be as yet undiagnosed with anorexia nervosa and revising our study recruitment materials to be more culturally sensitive increased the racial and ethnic diversity of our study participants who consented to be screened for eligibility.

However, as study recruitment continues, our data demonstrate that additional strategies are required to increase recruitment of other underrepresented racial groups, such as Black women. Mistrust of the medical and research community and apprehension about and lack of perceived benefit from research participation are considered key barriers to clinical research recruitment such populations [12, 13]. We hope to address these obstacles by developing strong partnerships with community health centers and cultural organizations that serve Black women and emphasizing that anorexia nervosa knows no racial boundaries. In addition, we are working to improve the diversity of our research team, as a more diverse workforce would help facilitate patient-centered research and may encourage individuals from minority groups to seek research opportunities [1, 2]. Institutional efforts, such as the Community Access, Recruitment, and Engagement (CARE) Research Center at Massachusetts General Hospital, that provides consultation services to study teams on enhancing community engagement and inclusion in research, as well as by institutional review boards, which can encourage diversity in clinical trial inclusion, may help address this issue more broadly.

The small sample size in the current clinical trial on bone health in women with anorexia nervosa makes it difficult to generalize our findings to other fields of clinical research in other ED populations. Differences in clinical trial design between our current and two previous clinical trials on bone health in anorexia nervosa may have contributed to the observed shift in participant demographics in our current trial. In addition, we did not study retention. Improving recruitment strategies is an important goal, but in addition, we need to ensure equitable retention with the goal of diverse representation across the life of a study. However, we hope that the rapid, tangible improvements we made in increasing the percent of non-White and Hispanic participants in our current clinical trial will serve as preliminary data for future studies on increasing racial and ethnic diversity in ED research. Although our focus was on increasing the representation of underrepresented racial and ethnic groups in our clinical research, improving participant diversity in ED clinical research in the future should also include diversity of other demographic variables such socioeconomic status, gender identity, and age.

In conclusion, barriers to diagnosis, treatment, and research recruitment of underrepresented groups with anorexia nervosa must be considered when designing clinical research studies in this disease, and strategies to address these barriers should be prioritized. All academic journals should require authors to report race and ethnicity data to (1) critically review the generalizability of study results, (2) identify factors that may contribute to differences in the development, maintenance, and treatment of EDs within underrepresented racial and ethnic groups, and (3) hold researchers accountable for improving diversity, equity, and inclusion in clinical research. We are hopeful that our experiences presented here will supplement the ongoing conversation about diversity in ED research and encourage further research into evidence-based recruitment strategies that address the historic underrepresentation of racially and ethnically diverse groups in ED research.

## Data Availability

The data that support the findings of this study are available from the corresponding author upon reasonable request.

## Abbreviations

ED: Eating disorder
RPDR: Research Patient Data Registry
